# Conjugation, characterization and toxicity of lipophosphoglycan-polyacrylic acid conjugate for vaccination against leishmaniasis

**DOI:** 10.1186/1423-0127-20-35

**Published:** 2013-06-03

**Authors:** Murat Topuzogullari, Rabia Cakir Koc, Sevil Dincer Isoglu, Melahat Bagirova, Zeynep Akdeste, Serhat Elcicek, Olga N Oztel, Serap Yesilkir Baydar, Sezen Canim Ates, Adil M Allahverdiyev

**Affiliations:** 1 Department of Bioengineering, Yildiz Technical University, Istanbul, Turkey; 2 Department of Biomedical Engineering, Yeni Yuzyil University, Istanbul, Turkey; 3 Department of Bioengineering, Firat University, Elazig, Turkey

**Keywords:** Polymer, Vaccine, Delivery, Adjuvant, Leishmania, Conjugation

## Abstract

Research on the conjugates of synthetic polyelectrolytes with antigenic molecules, such as proteins, peptides, or carbohydrates, is an attractive area due to their highly immunogenic character in comparison to classical adjuvants. For example, polyacrylic acid (PAA) is a weak polyelectrolyte and has been used in several biomedical applications such as immunological studies, drug delivery, and enzyme immobilization. However, to our knowledge, there are no studies that document immune-stimulant properties of PAA in Leishmania infection. Therefore, we aimed to develop a potential vaccine candidate against leishmaniasis by covalently conjugating PAA with an immunologically vital molecule of lipophosphoglycan (LPG) found in Leishmania parasites. In the study, LPG and PAA were conjugated by a multi-step procedure, and final products were analyzed with GPC and MALDI-TOF MS techniques. In cytotoxicity experiments, LPG-PAA conjugates did not indicate toxic effects on L929 and J774 murine macrophage cells. We assume that LPG-PAA conjugate can be a potential vaccine candidate, and will be immunologically characterized in further studies to prove its potential.

## Background

The research on the conjugates of synthetic polyelectrolytes with antigenic molecules, such as proteins, peptides, or carbohydrates, is an attractive area due to their highly immunogenic character compared to classical adjuvants [1,2]. In the last decade, the first vaccine based on this molecular structure was commercialized in Russia, and about 50 million people were vaccinated against influenza using the conjugate of polyoxidonium with protein subunits of influenza viruses [1].

The mechanism behind the immunogenic activity of polyelectrolyte-antigen conjugates, proposed by Kabanov [1], is based on the interaction of the conjugate with membrane proteins of immune-competent cells. According to the proposal, free polyelectrolyte chains of the conjugate clusters the membrane proteins and antigen linked with polyelectrolyte is presented to the receptor on the cell membrane. Clustering of membrane proteins changes the ion flux of the cell dramatically, and this serves as a signal to stimulate cellular mechanisms to enhance the immune response to antigen.

Poly (acrylic acid) (PAA) is a weak polyelectrolyte and has been used in several biomedical applications such as immunological studies [3], drug delivery [4], and enzyme immobilization [5]. Carboxylic acid groups of PAA enable further modifications, and for drug/biomolecule binding in mild conditions without any structural deterioration. It was determined that non-antigenic PAA, after being exposed to sheep erythrocytes, caused an increase in the immune response, where they acted as immunostimulants when injected into Balb/c mice [6]. Moreover, in this study researchers reported that low molecular weight synthetic polyelectrolytes did not show any activity in *in vivo* tests, implying that the effect becomes pronounced with increased molecular weight [7,8].

PAA has been used particularly as an adjuvant in veterinary vaccines. However, to our knowledge, there are no studies that document immune-stimulant properties of PAA in *Leishmania* infection. Leishmaniasis is still one of the world’s most neglected diseases, mainly in developing countries. Approximately 350 million people are considered at risk of contracting leishmaniasis, and nearly 2 million new cases occur yearly. Therefore, mortality and morbidity from leishmaniasis worldwide indicate a worrying increasing trend [9]. In general, conventional (killed or live attenuated parasites) and biotechnological methods have been studied for vaccine development against leishmaniasis, but unfortunately, there is still no effective vaccine.

Recently, lipophosphoglycan (LPG) has gained importance for vaccine studies. LPG is a crucial cell surface glycoconjugate of *Leishmania* parasites found on all surfaces including flagella. Lately, despite vaccine studies using LPG [10], the effective protection did not indicate improvement due to inefficient adjuvant. Numerous polymer-based vaccine studies have been carried out against many significant diseases such as HIV and influenza, and some of these are in clinical trial stage [11] or have been commercialized [1]. On the other hand, there are no polymer-based anti-leishmanial vaccine studies in the world to the best of our knowledge. Consequently, the aim of this study is to develop a potential vaccine candidate against leishmaniasis by covalently conjugating PAA with an immunologically vital molecule of LPG found in *Leishmania* parasites.

## Methods

### Materials

Polyacrylic acid (PAA), ethylenediamine, and NaCNBH_3_ were obtained from Aldrich (Schnelldorf, Germany). Water-soluble carbodiimide (EDC) was purchased from Sigma (Schnelldorf, Germany). Na_2_HPO_4_, NaH_2_PO_4_ and NaCl were obtained from Fluka (Schnelldorf, Germany). NaN_3_ was obtained from Applichem (Darmstadt, Germany). NaIO_4_ was obtained from Merck (Darmstadt, Germany). Ultra-pure water was obtained from a Millipore MilliQ system, which was used in all experiments and analyses.

### Parasite culture

*Leishmania donovani* (HOM/IN/83/AG83) promastigotes were cultured at 27°C in culture flasks as described previously [12]. The strain was kindly provided by Dr. Kwang-Poo Chang (RFUMS/The Chicago Medical School, USA). The growth of promastigotes was monitored every day using an inverted microscope (Olympus CK 40). The parasites were counted using a hemocytometer with a 20× objective under standard light microscopy.

### Scale up of *L. donovani* culture

*L. donovani* parasites grown in RPMI 1640 media were transferred into a 75 cm^2^ culture flasks with serum-free Brain Heart Infusion Medium (BHIM) including Hemin (Sigma, H9039), and Adenozin (Sigma, A9251). Parasite culture with a volume of 25–30 million parasites/mL was diluted by BHIM into a large-scale culture system. After 2 weeks, they were transferred into 250 mL glass made bottles on a shaker. 3–5 liters of *L. donovani* culture with a volume of 25–30 million parasites/mL were obtained. Continuous *L. donovani* culture was passaged once every two-weeks.

### Isolation and purification of LPG

All *Leishmania* sample pellets were collected and resuspended in 2 mL PBS. Equal volume of suspended pellets was transferred into corex tubes. The tubes were centrifuged at 4000 rpm at +4°C for 15 min. Chloroform-methanol was added to the tubes containing pellets at a 3.75-fold volume. Then, a sonication process was maintained for the tubes until whole pellet was resuspended, followed by incubation at room temperature for 1 h. Tubes were centrifuged at 4000 rpm at +4°C for 30 min. 4 mL of %9, 1-Propanol was added to the pellets in tubes. Sonication was applied to the tubes until the whole pellet was resuspended and then tubes were incubated at room temperature for 1 h, then centrifuged again. All supernatants were collected together and centrifuged at 14,000 rpm for 20 min. All supernatants were evaporated and following the evaporation, LPG extracts frozen at −40°C were centrifuged at 14,000 rpm for 15 min. In a 4-fold volume of supernatant, 0.1 M ammonium acetate was added onto the supernatant before loading it onto an octyl sepharose column. Column was packed with 5% - 60% 1-propanol/0.1 M ammonium acetate and fractions were collected (around 450 drops/tube).

### Conjugation of LPG and PAA

**Figure 1:**
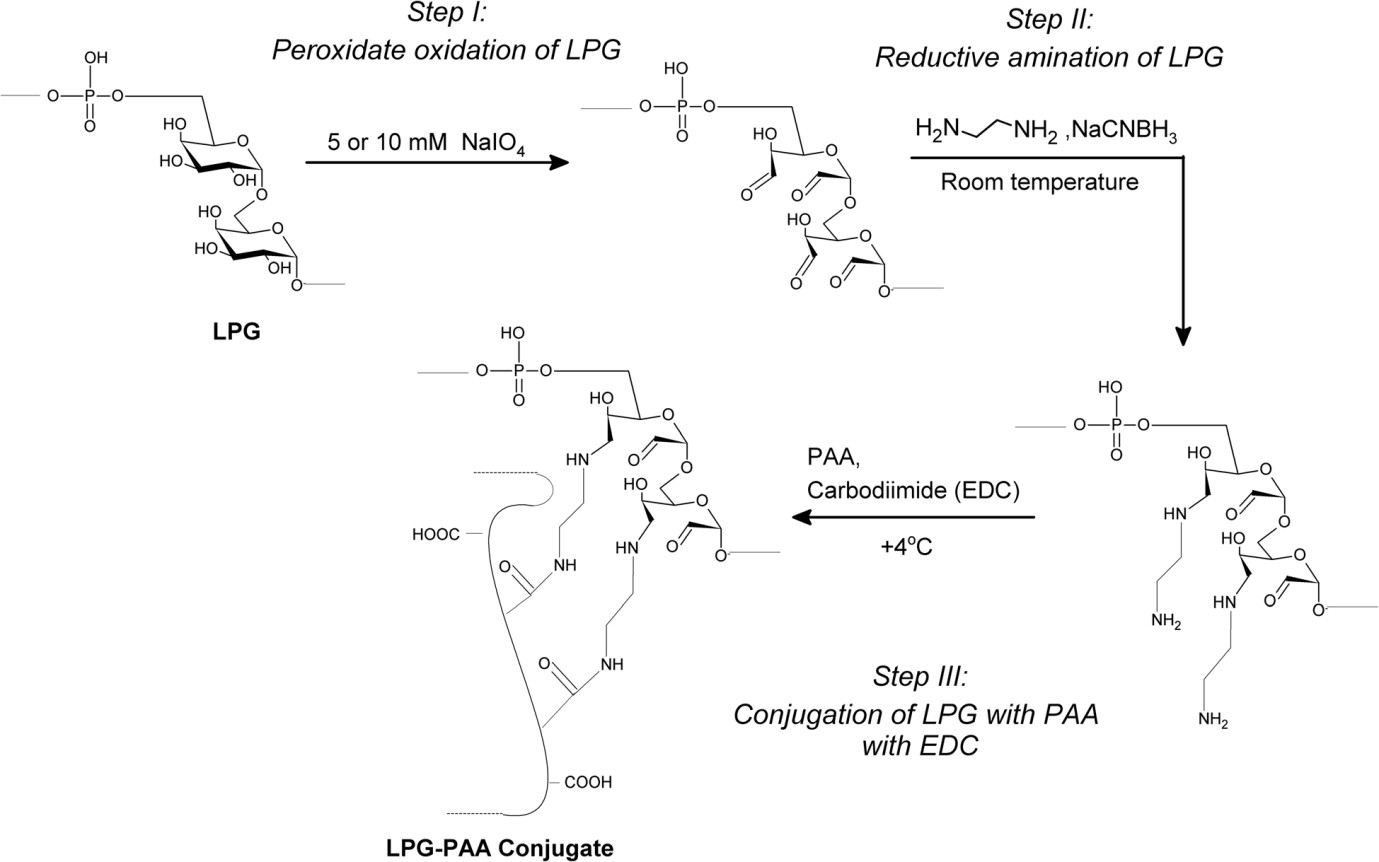
Conjugation mechanism of LPG with PAA.

A multi-step conjugation procedure (Figure 1) was followed to obtain LPG-PAA conjugates. In the first step, hydroxyl groups of LPG were modified to amino groups before the conjugation reaction. Hydroxyl groups of LPG were oxidized to aldehyde groups with 10 mM NaIO_4_ in 0.02 M phosphate buffer for 6 h. After the reaction, solution was dialyzed against water for 24 h at +4°C. Then, carbonyl groups of oxidized LPG were reductively aminated by the reaction with ethylenediamine and NaCNBH_3_ in 0.02 M phosphate buffer for 12 h. The reaction was followed by dialysis against 0.02 M phosphate buffer for 24 h at +4°C.

LPG and PAA were then conjugated at the concentration ratio of C_PAA_/C_LPG_ = 0.5 by using water-soluble EDC as a zero-length cross-linker. In this reaction, carboxylic acid groups of PAA (0.5 mg/mL) were activated with water-soluble EDC ([EDC]/[−COOH] = 1) at pH 5.0 for 30 min under vigorous stirring at room temperature. Then, aminated LPG, dissolved in the same volume of PAA solution, was added on activated PAA solution and the pH was adjusted to 7.0. Reaction solution was gently stirred overnight at 4°C.

This procedure was repeated by using 5 mM NaIO_4_ in oxidation step to minimize the modification in the structure, since any change in LPG molecule may alter its antigenic character. The final products, LPG-PAA conjugates, and individual PAA were characterized with Gel Permeation Chromatography (GPC) and MALDI-TOF MS. In mass spectroscopy, the conjugate chosen from the GPC results appropriate for cell studies was analyzed.

### GPC analysis

PAA and PAA-LPG conjugate were analyzed using gel permeation chromatography (GPC) with a triple detection system. Triple detection consists of refractive index (RI), right angle light scattering (LS), and viscosimetry (VIS) detectors, which were calibrated with PEO (22 kDa) standard solution. GPC analyses were performed with a Shimadzu Shim-Pack Diol-300 (50 × 0.79 cm) column at room temperature. PBS (pH = 7.1) was used for the mobile phase and flow rate was 1.0 mL/min.

### MALDI-TOF-MS analysis

Mass spectra of PAA, LPG and LPG-PAA conjugate were acquired on a Voyager-DE™ PRO MALDI-TOF mass spectrometer (Applied Biosystems, USA) equipped with a nitrogen UV-Laser operating at 337 nm. Spectra were recorded in reflectron mode with an average of 50 shots. Dithranol was used as MALDI matrix. Matrix and sample solutions were mixed to obtain W_sample_/W_matrix_ ratio of 1:10. 1 μL of matrix/sample mixture was deposited on the sample plate, dried at room temperature and analyzed.

### Cytotoxicity of PAA, LPG and PAA-LPG conjugates on J774 murine macrophages and L929 mouse fibroblastic cell line

J774 and L929 cells were seeded (10,000 cells/well) in 96-well flat bottom microplates with 100 μL of medium. The cells were allowed to attach to the bottom of the dish for 24 h at 37°C and then exposed to different concentrations of PAA, LPG, and PAA-LPG conjugates for 48 h. Afterwards, the cells were washed with PBS and incubated with 3-(4,5- dimethylthiazol-2-yl)-2,5-diphenyltetrazolium bromide (MTT) 100 μg/well for 4 h in the dark at 37°C. MTT solution was removed, and the cells were dissolved in 100 mL of dimethylsulfoxide and the absorbance measured in an ELISA reader at 540 nm (Bio-Rad). Each concentration was assayed in quintuplicate, and corresponding cell growth controls were used in each measurement. Each assay was also performed in triplicate.

### Flow cytometric measurements of apoptosis and necrosis by staining with Annexin-V/7-ADD

25 × 10^4^ L929 and J774 cells were cultured with RPMI 1640 (10% FCS) in 6-well plate. Cells were incubated at 37°C and 5% CO_2_ for 24 h. Cells were exposed to the LPG, PAA and LPG-PAA conjugate at 37°C and %5 CO_2_ for 48 h. Then cells were trypsinized and washed with PBS twice. 10 μl of Annexin V-FITC working solution, 5 μl 7-AAD and 100 μl binding solution were added and incubated in dark condition for 15 min. After then 900 μl binding solution were added each sample and analyzed with flow cytometry in 1 h.

## Results and discussion

### Conjugation of LPG and PAA

**Figure 2:**
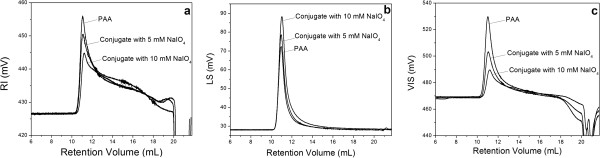
GPC chromatograms of PAA and LPG-PAA conjugates acquired from RI (a), LS (b) and VIS (c) detectors.

Isolated LPG was conjugated with PAA by a multi-step procedure. Final products were characterized first with GPC in order to identify if the conjugation occurred successfully. In addition, GPC with triple detectors gave detailed information about physicochemical properties of conjugates. GPC chromatograms of LPG-PAA conjugates prepared with 10 and 5 mM NaIO_4_ and individual PAA are shown in Figure 2, where PAA concentrations were equal in all solutions. LPG chromatograms are not shown in the figures since LPG did not elute from the column. We propose that LPG molecules were associated with the column material through the lipid chains.

In all PAA chromatograms obtained from different detectors, the peak eluting at 11.15 mL was attributed to PAA. LPG-PAA conjugates eluted at an identical retention volume as observed for PAA, however, peak areas of conjugates were less than PAA in RI chromatograms. RI signal of solutes is directly proportional to dn/dc and concentration. Change in RI area may be correlated to the dn/dc value of conjugates. The dn/dc values of polymers are ordinarily related to their chemical composition. Addition of LPG molecules into the PAA chain may have lowered the dn/dc value of PAA. Peak areas of conjugates in LS chromatograms increased in accordance with the increase in NaIO_4_ concentration used in oxidation of LPG, as seen in Figure 2b, and no other peaks were observed. LS signal is proportional to (dn/dc)^2^, concentration, and molecular weight of polymer. Increase in the LS signal can be correlated to an increase in the molecular weight of solute. In VIS chromatograms, peak areas of conjugates were significantly decreased compared to that observed for the PAA peak. By considering all the chromatograms together, we can say that LPG and PAA were successfully covalently conjugated.

Analysis of soluble polymeric systems with a multi-detector GPC system provides detailed information about physicochemical properties of particles, such as molecular weight, size, and conformation. In Table 1, physicochemical parameters of PAA and conjugates are shown, which were calculated from the peaks eluted approximately at 11.2 mL in GPC chromatograms. It is apparent from the increase in molecular weight values and changes in chromatograms that LPG was successfully conjugated to PAA, and molecular weight of conjugate increased in accordance with the modification in LPG structure. More amine groups formed by NaIO_4_ in the structure of LPG induced the number of bound LPG molecules on PAA, or the number of linked PAA chains on LPG molecule.

**Table 1:** Physicochemical parameters of PAA and LPG-PAA conjugates obtained from GPC analyses

**Sample**	**Mw (kDa)**	**Hydrodynamic Radius (nm)**	**a**
PAA	82.0	9.05	0.61
LPG-PAA conjugate (prepared with 5 mM NaIO4)	142.7	8.78	0.43
LPG-PAA conjugate (prepared with 10 mM NaIO4)	214.0	9.27	0.35

On the other hand, the size of the conjugate does not increase as much as the molecular weight. In addition, the size of the conjugate prepared with 5 mM NaIO_4_ is smaller than individual PAA and the conjugate synthesized with 10 mM NaIO_4_ is just 0.4 nm larger than PAA. Mark-Houwink constants, *a*, of conjugates are smaller than PAA, which indicates a more compact structure. The Mark-Houwink constant, *a*, is related to the conformation of polymers and varies from 0 (spherical) to 0.6-0.8 (random coil) to 2 (stiff chain). A more compact and dense structure was obtained after conjugation compared to that observed for the PAA chain. The compaction in conjugate structure can be explained by wrapping PAA more tightly around LPG via linking from more points on LPG. This phenomenon in conjugate size may also be explained by the hydrophobic effect of lipid chains in LPG molecules. Increase in the number of LPG molecules in a single conjugate might shorten the distance between lipid chains, and cause the compaction in size of conjugate by formation of hydrophobic cores.

**Figure 3:**
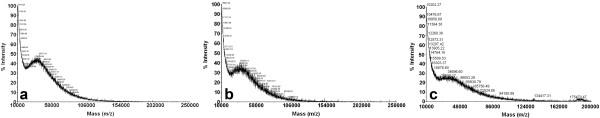
Mass spectra of PAA (a), LPG (b), and LPG-PAA conjugate (c) acquired from MALDI-TOF MS.

GPC results prove that LPG and PAA were successfully conjugated using both 5 mM and 10 mM NaIO_4_. However, modification in the structure of LPG can alter the antigenic behavior, so LPG-PAA conjugate prepared with a smaller amount of NaIO_4_ was chosen as the most favorable structure for further analyses in cell studies, and to keep the change in molecular structure to a minimum. Mass spectroscopy was chosen as the second method to verify the conjugate formation since it is a reliable, robust, and direct characterization technique. Mass spectra of LPG-PAA conjugate (prepared with 5 mM NaIO_4_), LPG, and PAA acquired from MALDI-TOF MS are shown in Figure 3. In MALDI-TOF mass spectrometry, only +1 charged molecular ions are formed and this phenomenon makes this method advantageous over other mass spectrometry techniques, such as electrospray ionization MS, in high molecular weight synthetic polymer analyses. The PAA used in this study is a polydisperse polymer, and a broad molecular mass distribution is observed in the mass spectrum (Figure 3a). Mass spectrum of LPG (Figure 3b) also represents a wide mass distribution. Number of phosphoglycan units in LPG structure varies [13,14] according to the growth stage, and this variation in the structure makes LPG a complex and polydisperse molecule. It is difficult to determine average molecular weights of polydisperse macromolecules in MALDI-TOF MS, since the ionization yield decreases with increasing chain length of macromolecules in most commercial MALDI-TOF detectors. In other words, high molecular weight species of polydisperse macromolecules do not ionize as much as low molecular weight chains [15]. Molecular masses having the highest intensity in mass spectra were assigned as average values for PAA and LPG, 40.3 and 35.6 kDA, respectively.

In the LPG-PAA mass spectrum, three mass distributions were observed as shown in Figure 3c. Mass distribution having maximum signal intensity at 34.6 kDa corresponds to unbound PAA or LPG molecules. Other mass distributions at 134.4 and 175.4 kDa were considered LPG-PAA conjugates having different quantities of components. Free and conjugated units may not be accurately quantified from these spectra due to the low ionization yield of higher molecular weight molecules, although the signal intensities of unbound units were much higher than conjugates’ signals. It is obvious from these spectra that signals observed at 134.4 and 175.4 kDa correspond exactly to LPG-PAA conjugation, and broad mass distribution at 34.6 kDa would be unbound LPG and PAA molecules.

In contrast to GPC chromatograms, free and conjugated molecules were observed simultaneously in the mass spectrum of LPG-PAA conjugate. The reason behind the co-eluting of free PAA and LPG-PAA conjugates in GPC may be due to the exclusion limit of GPC column packing. Molecules larger than the exclusion limit of the column elute at void volume and might prevent elution of unbound PAA and LPG-PAA conjugates separately in GPC analysis of LPG-PAA conjugate. As GPC and MS results are considered together, LPG and PAA were successfully conjugated, and conjugates having diverse structures were formed. LPG-PAA conjugate, prepared with 5 mM NaIO4 and analyzed with GPC and MS, was used in cell studies.

### Cell culture studies

**Figure 4:**
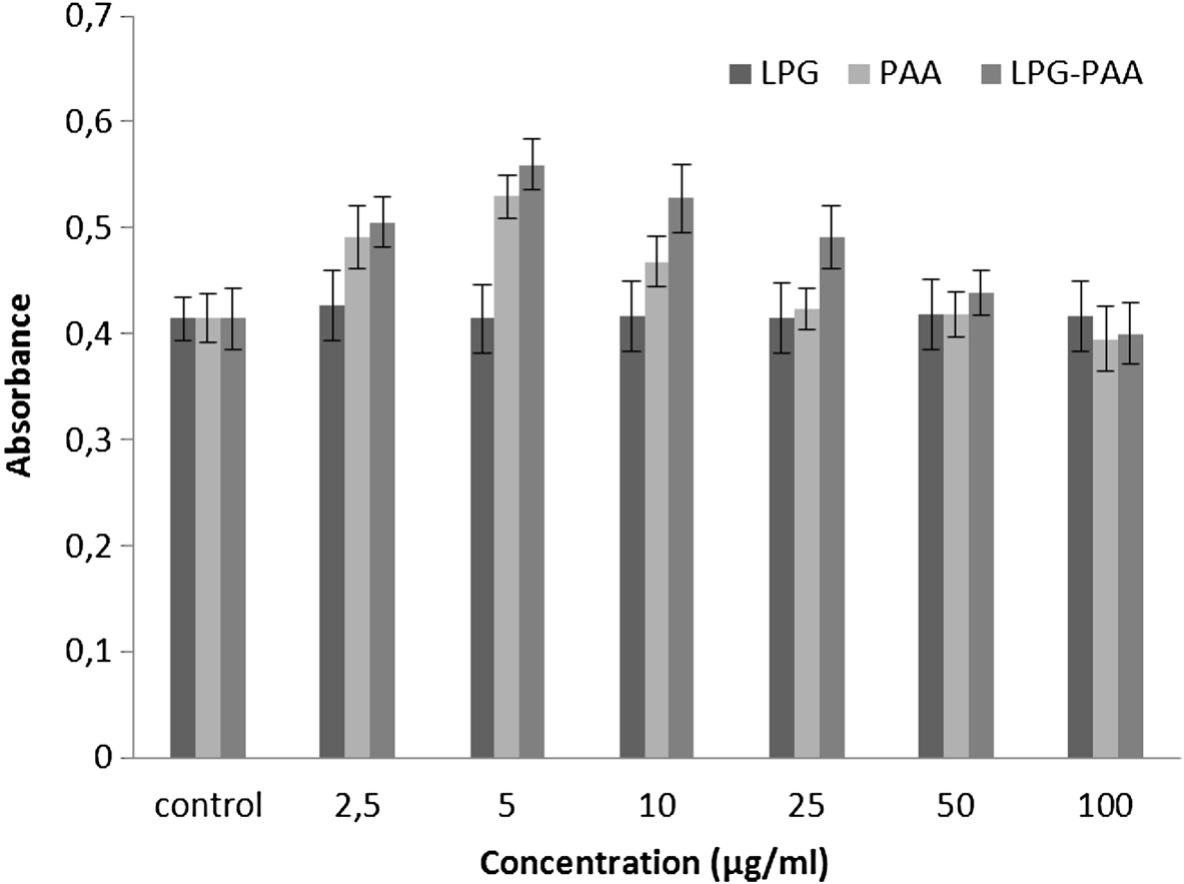
Cytotoxicity of LPG, PAA, and PAA-LPG conjugates on the J774 murine macrophages cell line.

**Figure 5:**
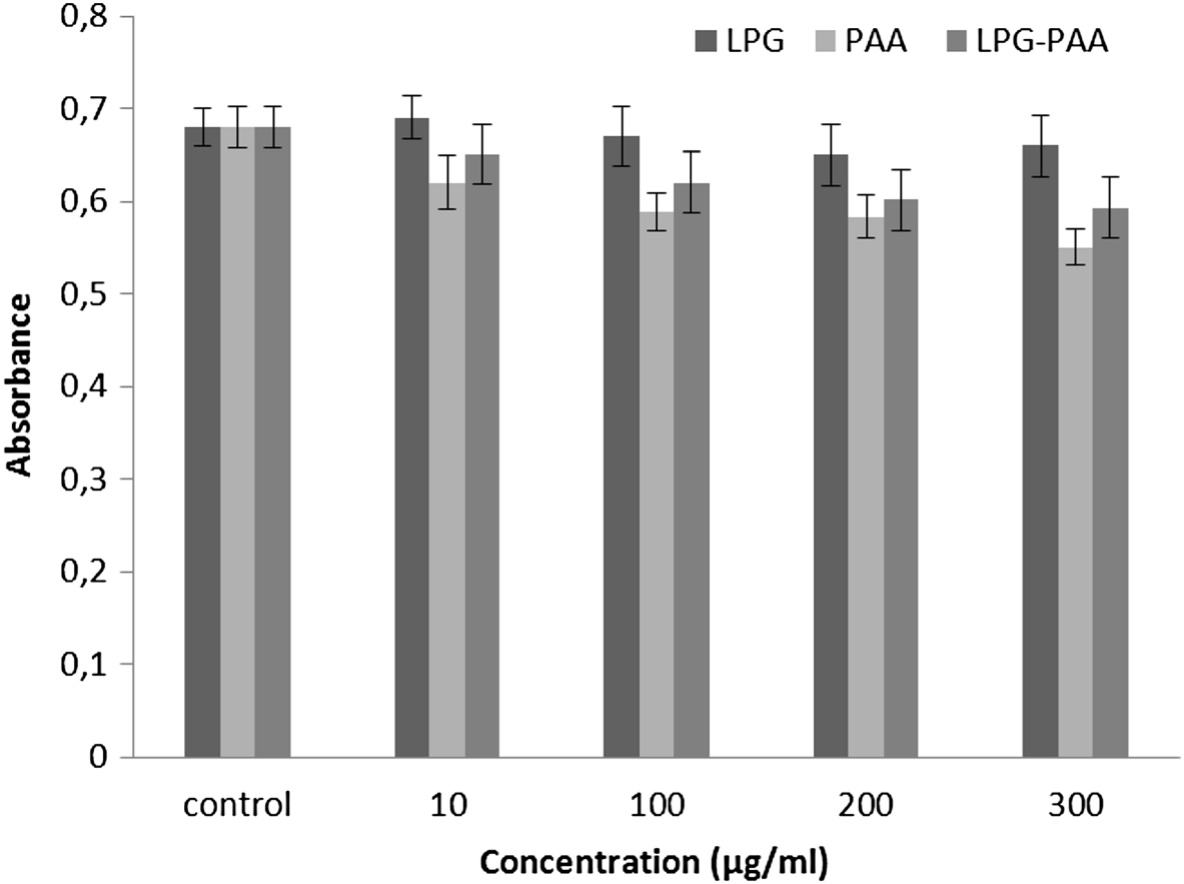
Cytotoxicity of PAA, LPG, and PAA-LPG conjugates on the L929 murine fibroblastic cell line.

The effect of PAA, LPG, and PAA-LPG conjugates with different concentrations on macrophages and fibroblasts were analyzed by MTT analysis, based on the ability of viable parasites to reduce the tetrazolium salt to an insoluble formazan product (Figures 4 and 5). As shown in Figures 4 (p = 0.068) and 5 (p = 0,077), there is no significant difference between PAA, LPG, and LPG-PAA conjugates. J774 and L929 cells indicated different absorbances depending on the viability, yet none of these concentrations of PAA, LPG, and LPG-PAA conjugates showed significant toxic effect on cells.

**Figure 6:**
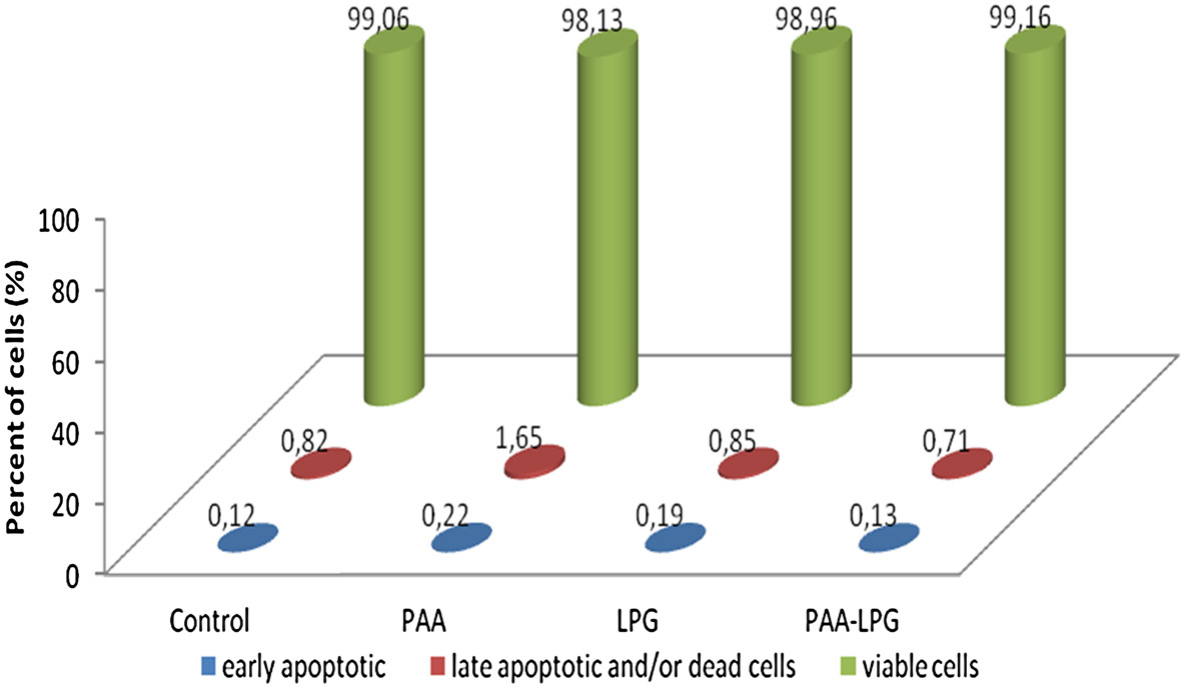
Percentages of early apoptotic, late apoptotic and/or dead cells and viable cells induced by LPG (35 μg/mL), PAA (70 μg/mL), and LPG-PAA conjugates (105 μg/mL) on the J774 murine macrophage cell line.

**Figure 7:**
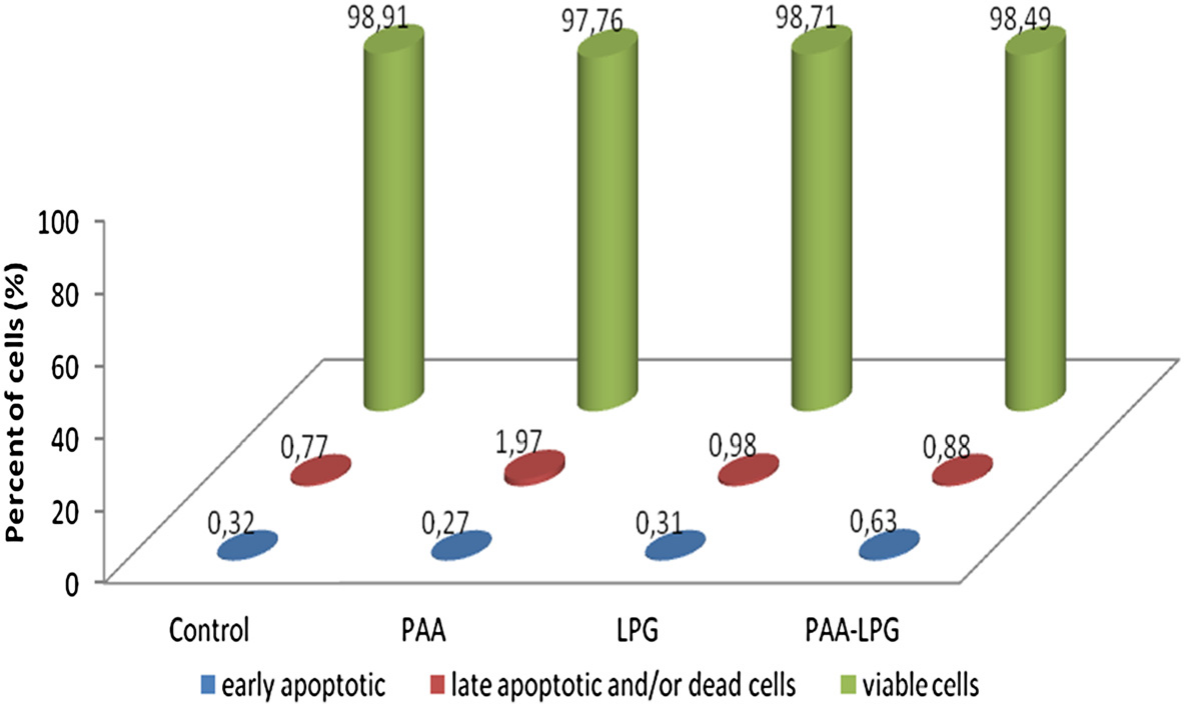
Percentages of early apoptotic, late apoptotic and/or dead cells and viable cells induced by LPG (35 μg/mL), PAA (70 μg/mL), and LPG-PAA conjugates (105 μg/mL) on the L929 mouse fibroblastic cell line.

The effects of PAA, LPG, and conjugate on cell viability and apoptosis are shown in Figures 6 and 7. There was no apoptosis or necrosis effect of PAA, LPG and conjugate on macrophage and fibroblast cell lines. There was no significant difference between groups and the control according to cell viability data for macrophages (p = 0.097) and fibroblasts (p = 0.113).

## Conclusion

LPG and PAA were conjugated using a multi-step procedure and analyzed with GPC and MS techniques. According to the results, LPG and PAA were successfully conjugated and conjugates presenting diverse structures were formed. In the synthesis procedure, the chemical structure of LPG was modified to obtain amine groups for conjugation, but this change in structure can alter antigenicity so that modification was kept minimal by lowering the oxidation agent (NaIO_4_) concentration. In cytotoxicity experiments, the LPG-PAA conjugate did not show toxic effects on L929 and J774 murine macrophage cells.

Synthetic polyelectrolytes such as PAA can dissociate in aqueous medium to form the polyanions and especially used as adjuvant in several viral infection studies [16-19]. Additionally, in one study, PAA was combined with sheep erythrocytes to enhance the immune response several fold [1,20]. The results obtained in this study may have relatively important implications in the development of anti-leishmanial vaccines, and amelioration of the immune response. Therefore, LPG-PAA conjugates can be vaccine candidates for leishmaniasis, and will be further developed in our future studies to prove their potential.

## Competing interests

The authors declare that they have no competing interests.

## Authors’ contributions

MT: conjugation of LPG and PAA, characterizations, drafting manuscript; RÇK: isolation of LPG, toxicity studies, drafting manuscript; SDİ, ZA: conjugation of LPG and PAA, reviewing manuscript; MB: isolation of LPG, toxicity studies, reviewing manuscript; SE, ONÖ, SYB, SCA: isolation of LPG, toxicity studies; AA: obtaining funding of the study and final approval of the manuscript. All authors read and approved the final manuscript.

## References

[B1] Kabanov VA From synthetic polyelectrolytes to polymer-subunit vaccines Pure Appl Chem 2004 76 1659 1678 10.1351/pac200476091659

[B2] Andrianov AK Marin A Roberts BE Polyphosphazene polyelectrolytes: a link between the formation of noncovalent complexes with antigenic proteins and immunostimulating activity Biomacromolecules 2005 6 1375 1379 10.1021/bm049329t 15877355

[B3] Basalp A Mustafaeva Z Mustafaev M Immunogenic Cu2 + −induced biopolymer systems comprising a steroid hormone, protein antigen, and synthetic polyelectrolytes Hybridoma 2002 21 45 51 10.1089/15368590252917638 11991816

[B4] Yuk SH Cho SH Lee HB pH-sensitive drug delivery system using OW emulsion J Control Release 1995 37 69 74 10.1016/0168-3659(95)00065-G

[B5] Bianchi D Golini P Bortolo R Cesti P Immobilization of penicillin G acylase on aminoalkylated polyacrylic supports Enzyme Microb Technol 1996 18 592 596 10.1016/0141-0229(95)00149-2

[B6] Diamantstein T Vogt W Ruhl H Bochert G Stimulation of DNA synthesis in mouse lymphoid cells by polyanions in vitro. I. Target cells and possible mode of action Eur J Immunol 1973 3 488 493 10.1002/eji.1830030807 4585312

[B7] Kabanov VA Mustafaev MI Nekrasov AV Norimov A Petrov RV Critical nature of the effect of the degree of polyelectrolyte polymerization on immunostimulating properties Dokl Akad Nauk SSSR 1984 274 998 1001 6610543

[B8] Petrov RV Kabanov VA Khaitov RM Mustafaev MI Norimov A Effect of heparin on the immunogenicity of electrostatic covalent albumin complexes with synthetic polyions. Immunogenicity of the triple covalent complex polyelectrolyte-protein-heparin Mol Gen Mikrobiol Virusol 1986 30 35 3785254

[B9] Desjeux P Leishmaniasis: current situation and new perspectives Comp Immunol Microbiol Infect Dis 2004 27 305 318 10.1016/j.cimid.2004.03.004 15225981

[B10] Pinheiro RO Pinto EF de Matos Guedes HL de Mattos KA Saraiva EM de Mendonça SCF Rossi-Bergmann B Protection against cutaneous leishmaniasis by intranasal vaccination with lipophosphoglycan Vaccine 2007 25 2716 2722 10.1016/j.vaccine.2006.05.093 16814903

[B11] Hanes J Cleland JL Langer R New advances in microsphere-based single-dose vaccines Adv Drug Deliv Rev 1997 28 97 119 10.1016/S0169-409X(97)00053-7 10837567

[B12] Allahverdiyev AM Koc RC Ates SC Bagirova M Elcicek S Oztel ON Leishmania tropica: the effect of darkness and light on biological activities in vitro Exp Parasitol 2011 128 318 323 10.1016/j.exppara.2011.04.002 21510933

[B13] Mahoney AB Sacks DL Saraiva E Modi G Turco SJ Intra-species and stage-specific polymorphisms in lipophosphoglycan structure control Leishmania donovani-sand fly interactions Biochemistry 1999 38 9813 9823 10.1021/bi990741g 10433687

[B14] Barron TL Turco SJ Quantitation of *Leishmania* lipophosphoglycan repeat units by capillary electrophoresis Biochim Biophys Acta, Gen Subj 2006 1760 710 714 10.1016/j.bbagen.2005.10.007 16310310

[B15] Montaudo G Montaudo MS Samperi F Montaudo G, Lattimer RP Matrix-assisted laser desorption ionization/mass spectrometry of polymers (MALDI-MS) Mass spectrometry of polymers 2002 Boca Raton: CRS Press LLC 431 446

[B16] Gelfi J Pappalardo M Claverys C Peralta B Guérin JL Safety and efficacy of an inactivated Carbopol-adjuvanted goose haemorrhagic polyomavirus vaccine for domestic geese Avian Pathol 2010 39 111 116 10.1080/03079451003604647 20390545

[B17] Hilgers L Nicolas I Lejeune G Dewil E Strebelle M Boon B Alkyl-esters of polyacrylic acid as vaccine adjuvants Vaccine 1998 16 1575 1581 10.1016/S0264-410X(98)00047-4 9711806

[B18] Krashias G Simon AK Wegmann F Kok WL Ho LP Stevens D Skehel J Heeney JL Moghaddam AE Sattentau QJ Potent adaptive immune responses induced against HIV-1 gp140 and influenza virus HA by a polyanionic carbomer Vaccine 2010 28 2482 2489 10.1016/j.vaccine.2010.01.046 20132920

[B19] Payne L Van Nest G Barchfeld G Siber G Gupta R Jenkins S PCPP as a parenteral adjuvant for diverse antigens Dev Biol Stand 1998 92 79 9554261

[B20] Petrov R Gvozdetskiĭ A Gorokhov A Evdakov V Kabanov V Study of the mechanisms of the action of heparin and poly-4-vinylpyridine on immunogenesis Zh Mikrobiol Epidemiol Immunobiol 1974 37 4615519

